# Brain morphology mediating the effects of common genetic risk variants on Alzheimer’s disease

**DOI:** 10.1177/25424823251328300

**Published:** 2025-03-24

**Authors:** Esmee M Breddels, Yelyzaveta Snihirova, Ehsan Pishva, Sinan Gülöksüz, Gabriëlla AM Blokland, Jurjen Luykx, Ole A Andreassen, David EJ Linden, Dennis van der Meer, For the Alzheimer's Disease Neuroimaging Initiative

**Affiliations:** 1 Department of Psychiatry and Neuropsychology, Faculty of Health, Medicine and Life Sciences, School for Mental Health and Neuroscience, Maastricht University, Maastricht, The Netherlands; 2 Faculty of Health and Life Sciences, Medical School, University of Exeter, Exeter, UK; 3 Department of Psychiatry, Yale University School of Medicine, New Haven, CT, USA; 4 Department of Psychiatry, Amsterdam University Medical Center, Amsterdam, the Netherlands; 5 GGZ in Geest Mental Health Care, Amsterdam, The Netherlands; 6 Centre for Precision Psychiatry, Division of Mental Health and Addiction, Oslo University Hospital & Institute of Clinical Medicine, University of Oslo, Oslo, Norway; 7 KG Jebsen Centre for Neurodevelopmental disorders Research, Oslo University Hospital & University of Oslo, Oslo, Norway

**Keywords:** Alzheimer's disease, Alzheimer's Disease Neuroimaging Initiative, apolipoprotein E4, brain morphology, genetic variation, mediation analysis, neuroimaging, UK Biobank

## Abstract

**Background:**

Late-onset Alzheimer's disease (LOAD) has been associated with alterations in the morphology of multiple brain structures, and it is likely that disease mechanisms differ between brain regions. Coupling genetic determinants of LOAD with measures of brain morphology could localize and identify primary causal neurobiological pathways.

**Objective:**

To determine causal pathways from genetic risk variants of LOAD via brain morphology to LOAD.

**Methods:**

Mediation and Mendelian randomization (MR) analysis were performed using common genetic variation, T1 MRI and clinical data collected by UK Biobank and Alzheimer's Disease Neuroimaging Initiative.

**Results:**

Thickness of the entorhinal cortex and the volumes of the hippocampus, amygdala and inferior lateral ventricle mediated the effect of *APOE* ε4 on LOAD. MR showed that a thinner entorhinal cortex, a smaller hippocampus and amygdala, and a larger volume of the inferior lateral ventricles, increased the risk of LOAD as well as vice versa.

**Conclusions:**

Combining neuroimaging and genetic data can give insight into the causal neuropathological pathways of LOAD.

## Introduction

Late-onset Alzheimer's disease (LOAD) is a progressive, neurodegenerative disease with a complex polygenic architecture^1^ and an estimated broad heritability of 60–80% based on twin studies.^2^ A diagnosis of LOAD is primarily based on the presence of clinical symptoms^3^ such as memory impairment and disturbances in mood and behavior. Available medications only slow down progression and relieve symptoms at best.^3,4^ The discovery of effective disease-modifying treatments is complicated by the late diagnosis, when neurons have already degenerated to the point where the process is irreversible.^5–7^ However, the pathology of LOAD starts before the onset of clinical symptoms and the presence of biomarkers such as neurodegeneration could potentially be used for an earlier diagnosis. The use of biomarkers for an early diagnosis is currently difficult because pathological hallmarks such as amyloid-β and tau are not specific enough to distinguish LOAD from normal ageing and are mainly used in research settings.^8–10^ In addition, common genetic variants found by genome-wide association studies (GWAS)^11–13^ had only small effect sizes and, even combined, explained only a small fraction of the heritability of LOAD.^11^ The strongest genetic effect is seen for *APOE*, explaining 27.3% of the heritability.^14^ *APOE* ε4 status is thereby one of the most common risk factors of LOAD and is used for stratification of populations in the clinical and research setting, but the presence of *APOE* ε4 is not enough to make a clinical diagnosis of LOAD.^15^

LOAD is characterized by neurodegeneration and has been associated with alterations in the structure of numerous cortical and subcortical brain regions.^16^ The progression of neurodegeneration in LOAD follows a distinct pattern, starting in the middle temporal lobe, the entorhinal cortex and the hippocampus.^17–19^ After, neurodegeneration spreads to other brain regions, such as the frontal areas. Presumably, only a subset of brain regions is involved in the core pathophysiology, whereas the remainder is affected by and involved in secondary disease processes. There are many hypotheses about LOAD's primary pathology, mostly regarding accumulation of amyloid-β and tau pathology.^20^ Brain volume loss occurring later in the disease, such as that of frontal areas, might be the result of disruption of major signaling pathways due to the degeneration of the middle temporal lobe,^18,21^ but could also be induced by the same processes that induced neurodegeneration in the entorhinal cortex and hippocampus.^20^

Neuroimaging techniques, genetics, and especially the combination of the two have been underutilized as tools for predicting both the occurrence and progression of LOAD.^22–24^ Coupling genetic determinants of LOAD with measures of brain morphology could localize and identify primary causal neurobiological pathways. The aim of the present study was to find causal pathways from genetic risk variants via brain morphology to LOAD. To accomplish this, mediation analysis and Mendelian randomization (MR) were performed. First, we tested the association between the genetic variants and LOAD. Next, we aimed to estimate whether genetic risk variants of LOAD altered the morphology of certain brain regions, and whether this change in morphology, was related to LOAD.

## Methods

### Study population

To determine which brain regions mediate the effect of LOAD genetic risk factors, we performed mediation analysis using data from the UK Biobank (UKB) population cohort. To validate our findings, all analyses were repeated on a second cohort, namely the case-control cohort of the Alzheimer's Disease Neuroimaging Initiative (ADNI).

The UKB consortium has collected genetic, lifestyle and health information of approximately 500,000 individuals aged 40 to 69 years at time of recruitment.^25^ A subgroup of participants was reinvited to a follow-up visit which included brain magnetic resonance imaging (MRI) scanning.^26^ The dataset used in the current study included 46,852 participants of whom brain MRI scans were available (Supplemental Figure 1). We selected individuals who had been genotyped (n = 45,549) and were of white British ancestry (n = 39,565), as determined by self-report and confirmed by genetic principal components (field 22006 of the UKB dataset). Only individuals with white British ancestry were included because the UKB release 2b 10,000 European reference panel population was used to obtain the genetic information. The same inclusion criteria and reference panel was used by Jansen et al.^11^ to obtain the GWAS summary statistics used in this study. We excluded participants without parental information on LOAD status, age or age at death (n = 643). Participants with missing genotypes for specific SNPs of interest were excluded in a model-specific manner. The final sample size ranged between 37,728 and 38,922 per analysis (Supplemental Table 2).

The ADNI cohort (http://adni.loni.usc.edu/) is a public-private partnership with the primary goal of ADNI to test whether serial MRI, positron emission tomography (PET), other biological markers, and clinical and neuropsychological assessment can be combined to measure the progression of mild cognitive impairment (MCI) and early AD. The ADNI sample, containing data from ADNI1, ADNIGO and ADNI2, had a sample size of 1740 participants. Exclusion for the current study was based on the absence of MRI data (n = 672) or genotyping (n = 279), American Indian or Alaskan Native, Asian or more than one ancestry (n = 9), or a diagnosis of MCI (n = 304). The final sample contained 476 subjects, of which 223 were LOAD cases and 253 were cognitively normal controls (Supplemental Figure 1).

A description and the corresponding UKB and ADNI field code(s) of all the variables used for this study are presented in Supplemental Table 1. The data was provided by the respective consortia. The UKB consortium obtained ethical approval from the North West Multi-centre Research Ethics and ADNI obtained ethical approval from the Ethical Committees of each institution where the work was performed.^27^ No extra regulations were required for the current study.

### Ascertainment of late-onset Alzheimer's disease

Our sample obtained from UKB contained only a few participants were diagnosed with LOAD (ICD code G30 and/or F00). Therefore we created a LOAD proxy score for the UKB, in accordance with previous genetic studies.^11,12,28^ To create the LOAD proxy score, information on parental LOAD status and age (at death) was used. Parental information was self reported in UKB by the participants. The LOAD proxy score reflected the number of biological parents affected by LOAD (0, 1, or 2). Unaffected parents might not have passed the period of risk and might still get the disease or deceased before developing the disease. To account for this risk, a contribution to the LOAD proxy score was based on the age (or age at death) of the parent (weight = (100-age)/100). The maximum contribution of a parent without LOAD was set to 0.32, the maximum population prevalence for AD, based on the prognosis of Hebert et al.^11,29^ For more information on the calculation of the LOAD proxy score, see Jansen et al.^11^

The mean (±SD) LOAD proxy score in the UKB sample was 0.66 (±0.41). The distribution of the LOAD proxy score did not follow a normal distribution. A simulation with 100,000 replications concluded that the type one-error rate was preserved (Supplemental Material), and linear regression models were appropriate.

The ADNI cohort contained memory-related diagnostic information, categorized as LOAD, MCI or cognitively normal, based on Mini-Mental State Examination (MMSE), Clinical Dementia Rating (CDR) and NINCDS/ADRDA Alzheimer's criteria.^30^ The most recent diagnosis available per subject was used and only LOAD cases and cognitively normal controls were included, excluding participants with MCI.

### Genotyping

Detailed information on the genotyping process of UKB^31^ and ADNI (website: https://adni.loni.usc.edu/data-samples/data-types/genetic-data/) have been reported elsewhere. Briefly, 488,377 UKB participants were genotyped for 805,426 markers using the UK BiLEVE Axiom Array (49,950 participants) and the UKB Axiom Array (438,427 participants). We made use of the UKB v3 imputed data, based on the Haplotype Reference Consortium (HRC) reference panel. The BGEN file obtained from UKB was converted to PLINK binary format. SNPs with more than 10% missingness and SNPs failing the Hardy–Weinberg equilibrium test at p < 1 × 10−9 were filtered out.

Genotype data from the ADNI sample were obtained from the ADNI LONI database. Whole genome genotyping was performed using different arrays depending on the phase of recruitment. The Illumina Human610 Quad Beadchip and Illumina HumanOmniExpress Beadchip were used for ADNI1 and ADNIGO/2, respectively. The 1000 Genomes Phase 3 reference panel was used for imputation. Based on qualitative assessment of clustering of the first two principal components, non-European cases were removed. Quality control (QC) and imputation processes were performed according to published recommendations.^32^ Briefly, QC included removal of individuals with more than 10% missingness and variants deviating from Hardy-Weinberg equilibrium <1e−3, or with low imputation quality (r^2 ^< 0.3).

### Selection of genetic risk variants

Our initial analysis was performed on UKB, therefore UKB specific GWAS summary statistics on the LOAD proxy score were obtained from Jansen et al.^11^ and used as input for the SNP2GENE function in the online platform Functional Mapping and Annotation of Genome-Wide Association Studies (FUMA), using default settings for the clumping.^33^ Based on the UKB release 2b 10,000 European reference panel population and UKB specific GWAS summary statistics, 13 genomic risk loci were identified and the lead SNP for each locus was selected (Supplemental Table 2). Genotype information on these 13 lead SNPs of interest was extracted for all participants. No information could be obtained for rs115674611, rs184384746 and rs187370608 in either UKB or ADNI. The lack of genotype information on these SNPs might be due to differences in the filters used by Jansen et al.^11^ and the current study. Analyses were performed on the remaining 10 SNPs and *APOE* ε4 status. To calculate the number of *APOE* ε4 alleles per participant, information on rs429358 and rs7412 was extracted for each participant. The nucleotide at rs429358 and rs7412 can be either cysteine (T) or arginine (C), and their combination leads to four *APOE* alleles: ε1, rs429358 (C) + rs7412 (T); ε2, rs429358 (T) + rs7412 (T); ε3, rs429358 (T) + rs7412 (C); and ε4, rs429358 (C) + rs7412 (C) as described previously.^34^

### MRI data acquisition and pre-processing

T1-weighted MRI scans for UKB were acquired at three different sites, all using identical 3 Tesla Siemens Skyra scanners (Siemens Healthineers, Erlangen, Germany), with a 32-channel head coil and identical protocols.^26^ Images were sent to the imaging center, where they were reconstructed and pre-processed using an automated pipeline. Pre-processing included corrections for head motion and other artefacts, and automated quality control for identifying equipment issues such as coil failure. Detailed information on the MRI acquisition and processing was published elsewhere.^35^

ADNI T1-weighted MRI protocols can be found at http://adni.loni.usc.edu/methods/documents/mriprotocols/. In short, all scans were acquired using MRI scanners from GE Healthcare, Philips Medical Systems, or Siemens Medical Solutions. Images were sent to the Mayo Clinic for data correction and quality control procedures.

The current study included brain morphology measures, made available by UKB and ADNI, of surface area and mean thickness of 34 cortical regions, and volume of 25 subcortical, ventricular, callosal and other automatically segmented non-cortical regions. Cortical reconstruction and volumetric segmentation for all images (UKB and ADNI) was performed using FreeSurfer software,^36^ versions v5.3 (UKB) and v5.1 (ADNI1/2/GO). Measures from the left and right hemisphere were summed for measures of surface area and volume and averaged for thickness. In addition, intracranial volume (ICV), total surface area and global mean thickness were extracted or calculated. ICV was readily available for both UKB and ADNI, whereas total surface area and global mean thickness could only be extracted for UKB. For ADNI participants, total surface area was calculated as the sum of the surface areas of the 34 cortical regions included in the study. The global mean thickness was a weighted sum of the mean thickness of the 34 cortical regions, where the contribution of each region was determined by the proportion of the region's surface area of the total surface area. All brain measures were mean-centered and standardized, i.e., converted to Z-scores, before inclusion in the models.

### Statistical analysis

All analyses were performed in R version 4.1.2.^37^ Data from ADNI were retrieved via the *ADNIMERGE* package^38^ obtained from (https://ida.loni.usc.edu/login.jsp).

### Mediation analysis

**Figure 1.**
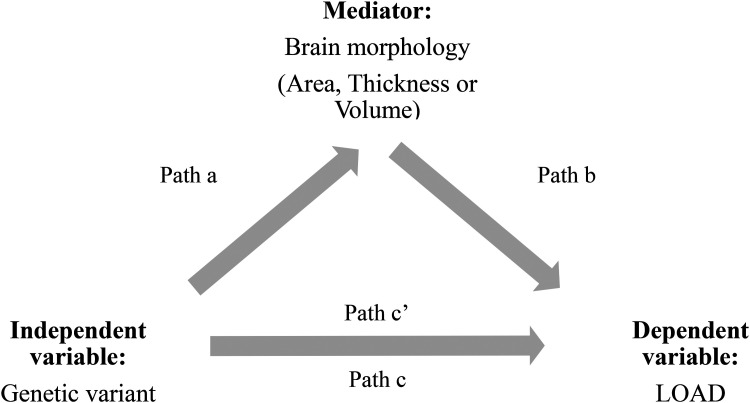
Diagram of the mediation model. Path a represents the association between the genetic variant and measure of brain morphology (mediator model). Path b represents the association between brain morphology and late-onset Alzheimer's disease (LOAD). Combining path a and path b gives the mediation effect. Path c represents the total effect of the genetic variant on LOAD. Path c’ is the indirect effect between the genetic variant and LOAD that remains after correcting for the mediation effect of brain morphology.

The relationship between genetic variation, brain morphology and LOAD was assessed using mediation analysis (Figure 1).^39,40^ First, we calculated the total effect of the genetic variant on LOAD (path c). Next, we tested the relation between the genetic variant and brain morphology (path a). The total effect is composed of the direct effect of the genetic variant on LOAD (path c’) and the indirect or mediation effect via brain morphology (path a*b). The estimate of the mediation effect is the product of path “a” and path “b” and significance was determined through bootstrapping, implemented in the *mediation* package^41^ in R. All models included age and sex as covariates. Covariate selection was based on the directed acyclic graph (DAG)^42^ presented in Supplemental Figure 2. In addition, to correct for differences in brain size, a global measure was included in all mediation models. The global measure included was either total surface area, global mean thickness or total intracranial volume, depending on the type of brain measure included as mediator (surface area, cortical thickness or volume respectively). Lastly, all models on the UKB sample included assessment site as a covariate. Due to the large number of assessment sites for ADNI and the small sample size per site, site could not be included as a covariate, in line with previous studies using this data.^27,43,44^ ADNI has standardized MRI protocols to minimize potential site and scanner effects, and no significant variability of MRI performance between sites has been previously shown.^27^

The analyses resulted in 1023 models for UKB and 1023 models for ADNI. Multiple comparisons correction was performed using the false discovery rate (FDR) set to 5%. Brain measures as well as the genetic variants were correlated, the formula for FDR was adjusted by Li and Ji^45^ to use the effective number of independent tests rather than the total number of tests. The adjusted formula was used to calculate an adjusted alpha value that was used as the significance threshold for each test.

Combining the results from our analysis on UKB and ADNI can potentially reveal new associations which might be borderline non-significant in the individual analysis. Therefore, the mediation effects obtained from the analysis on UKB and on ADNI were pooled using the inverse-variance weighted (IVW) method for meta-analysis.^46^ The standard errors of the mediation analysis estimates were approximated using the width of the 95% confidence intervals obtained via bootstrapping (5000 bootstrap simulations).

### Mendelian randomization

One-sample bidirectional MR was used to determine the direction of causality, whether the change in brain morphology is cause or consequence of LOAD. The rational of MR is that if a change in brain morphology causes LOAD, any variable influencing brain morphology should also influence the risk of LOAD.^47,48^ MR is based on the instrumental variable assumptions stating that: (1) the genetic variants (instrumental variables) are associated with brain morphology (exposure), (2) the genetic variants are not associated with LOAD (outcome) due to confounding pathways, and (3) the genetic variants do not affect the outcome except potentially via brain morphology.^47,48^ The instrumental variables for this study will include the same 10 SNPs (see Supplemental Table 2) and *APOE* ε4 status as the mediation analysis. The selection of the genetic variants is described above. Only brain measures that were significant mediators were used as exposures. The same covariates as for the mediation analysis were used.

MR was performed using the *MendelianRandomization* package^49^ in R. To minimize the risk of bias due to violation of assumptions, several MR methods were applied and compared for consistency of results. The MR methods used were the inverse variance weighted (IVW) method^50^ (robust when all genetic variants are valid and horizontal pleiotropy is absent), weighted median^51^ (robust to violations of assumptions by maximum half of the instrumental variables and outliers), and MR-Egger^52,53^ (robust to violation of the horizontal pleiotropy assumption by all instrumental variables). The validity of the results was additionally assessed by the intercept of the MR-Egger method (measure of pleiotropic effects^52^), I2 statistic (measure of instrument strength MR-Egger^51,52^), Cochran's Q statistic (measure of heterogeneity between variant-specific causal estimates^48^), and leave-one-out analysis (assessing the contribution of each genetic variant to the MR analysis^48^). The results of the IVW method were presented in the main text. To correct for testing multiple brain regions, the results were adjusted using FDR correction set to 5%.^54^

## Results

### Demographics

The UKB sample of 38,922 participants had a mean (±SD) age at the time of MRI of 64.4 (±7.7) years and the distribution over males and females was 47.7% and 52.3% respectively (Table 1). The mean (SD) LOAD proxy score was 0.64 (±0.40) for males and 0.67 (±0.41) for females. In the ADNI dataset, the group of LOAD cases had more males (62.3%) compared to the control group (46.1%) (Table 1). The mean age (±SD) was 76.1 (±7.7) years in the LOAD group and 76.2 (±6.8) years for controls.

**Table 1.** Demographics of the UK biobank (UKB) and ADNI participants. The mean and standard deviation (SD) of age and the number and percentage of male and female participants (n) in the two cohorts was shown.

	UKB	ADNI	
		Cases	Controls
Age (y (SD))	64.4 (7.7)	76.1 (7.74)	76.2 (6.79)
Male (n (%))	18,552 (47.7%)	129 (52.7%)	116 (47.3%)
Female (n (%))	20,370 (52.3%)	94 (40.7%)	137 (59.3%)

### Associations between genetic variants and LOAD (path c)

*APOE* ε4, rs75627662, and rs7384878 were significantly associated with LOAD in both UKB and ADNI. Five additional SNPs were significantly related to LOAD in UKB participants. No additional SNPs were significantly associated with LOAD diagnosis in ADNI. The identified associations are summarized in Supplemental Table 3.

### Associations between genetic variants and brain measures (path a)

**Figure 2.**
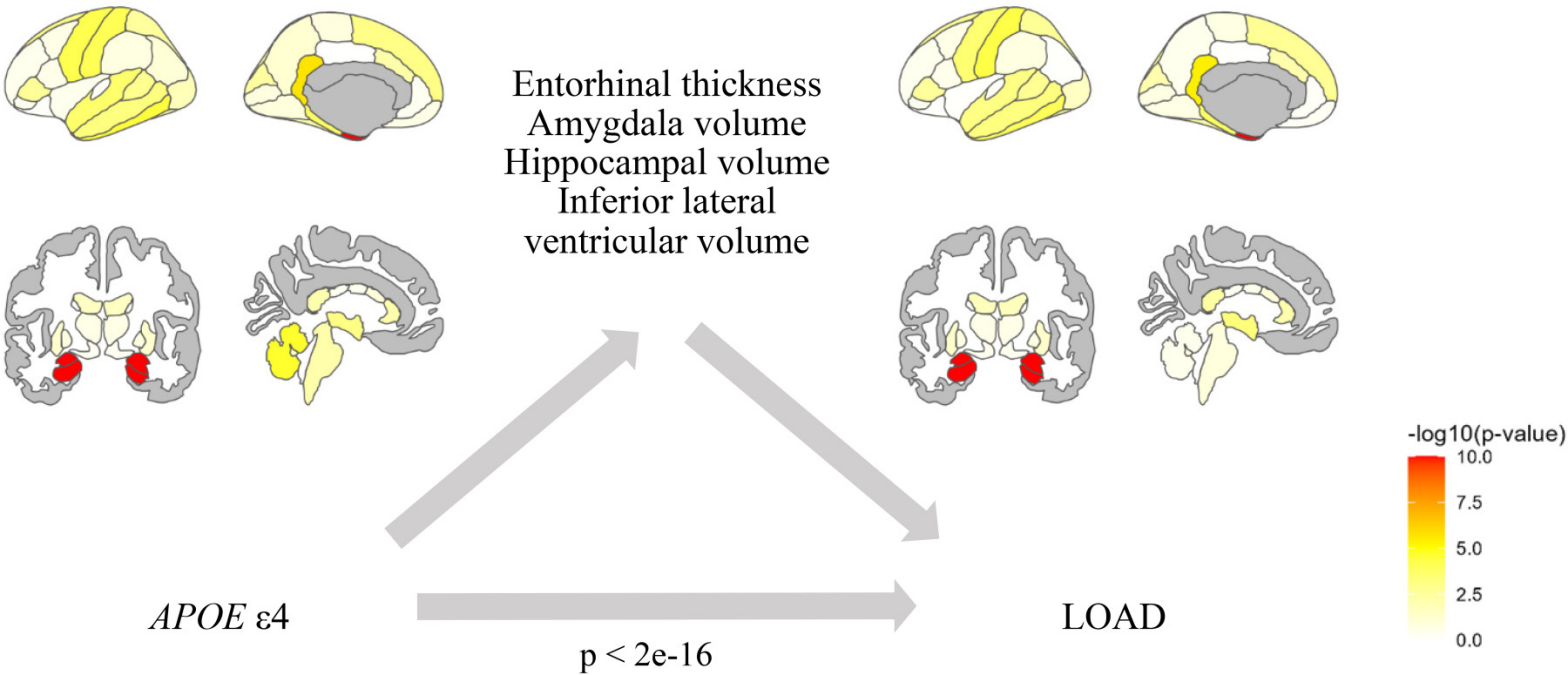
Mediation diagram depicting the results of the mediation analysis from *APOE* ε4 to brain morphology to late-onset Alzheimer's disease (LOAD) obtained in the ADNI sample. The effect of *APOE* ε4 was significantly mediated by the entorhinal cortex, amygdala, hippocampus and inferior lateral ventricle. The color coding indicates significance of the mediation test, as indicated in the legend.

Analysis on UKB data revealed 29 associations between genetic risk factors and brain regions (Supplemental Table 4). Most associations were found for *APOE* ε4, which was related to 9 brain measures. In the ADNI subset, significant associations of *APOE* ε4 with the mean thickness of the entorhinal cortex, and the volumes of the amygdala, hippocampus and inferior lateral ventricle were found (Figure 2, Table 2). Additionally, there was a significant association between rs75627662 and entorhinal thickness, amygdala and hippocampal volume, and between rs7384878 and putamen volume.

**Table 2.** Subset of significant results from all mediation analysis performed. The mediator model (path a) represents the effect of the genetic variant on the brain measure. The mediation effect (a*b) is the indirect of mediation effect of the brain measure on the association between the variant and late-onset Alzheimer's disease. *indicates the p-value is smaller than the FDR threshold, thus significant.

			Mediator model (path a)			Mediation effect (a*b)		
Genetic					FDR			FDR	
Variant	Brain measure		Estimate	p	threshold	Estimate	p	threshold	
APOE4	Thickness	Entorhinal	−0.333	3.7E−09	0.0002*	0.085	<2E−16	0.0003*
	Volume	Amygdala	−0.409	2.5E−10	0.0001*	0.131	<2E−16	0.0003*
		Hippocampus	−0.424	4.2E−11	0.0001*	0.140	<2E−16	0.0003*
		Inferior lateral ventricle	0.317	1.6E−06	0.0002*	0.109	<2E−16	0.0003*
rs75627662	Thickness	Entorhinal	−0.246	1.5E−05	0.0003*	0.068	<2E−16	0.0003*
	Volume	Amygdala	−0.242	0.0003	0.0004*	0.079	<2E−16	0.0003*
		Hippocampus	−0.291	1.0E−05	0.0003*	0.111	<2E−16	0.0003*
rs7384878	Thickness	Superior parietal**	0.019	3.4E−06	0.0003*	0.0003	<2E−16	0.0001*
	Volume	Putamen	0.248	0.0001	0.0004*	−0.042	<2E−16	0.0003*

*Significant.

**Effect found in UK Biobank; all other effects were detected in ADNI.

### Mediation effect of brain measures (path a*b)

In the UKB sample, we found a single mediation effect: the effect of rs7384878 on the LOAD proxy score was mediated by the thickness of the superior parietal cortex. A cytosine at rs7384878 compared to thymine was associated with a thicker superior parietal cortex, mediating the association between rs7384878 and an increased LOAD proxy score.

In ADNI, brain regions that were significantly related to genetic variants in path *a*, were also the mediators of the effect of the genetic variant on LOAD (Figure 2). Entorhinal thickness and the volumes of the amygdala, hippocampus and inferior lateral ventricle significantly mediated the effect of *APOE* ε4 on LOAD (Table 2). Entorhinal thickness, hippocampal and amygdala volume additionally mediated the effect of rs75627662 on LOAD. The volume of the putamen mediated the effect of rs7384878 on LOAD. Looking at the directionality of the effects, *APOE* ε4 and rs75627662 were associated with thinner entorhinal cortex and smaller volumes of the amygdala and hippocampus, relating to a higher risk of LOAD. *APOE* ε4 allele count was associated with larger volume of the inferior lateral ventricles and associated with a higher risk of LOAD. Rs7384878 was associated with a higher volume of the putamen and decreasing risk of LOAD.

Combining the effects in the UKB and the ADNI by means of meta-analysis did not result in any significant effect (Supplemental Table 6). Closer inspection of the results showed that mediation effects that were significant in one cohort, were highly non-significant or in opposite direction in the other cohort, see Supplemental Table 4.

### Mendelian randomization

**Figure 3.**
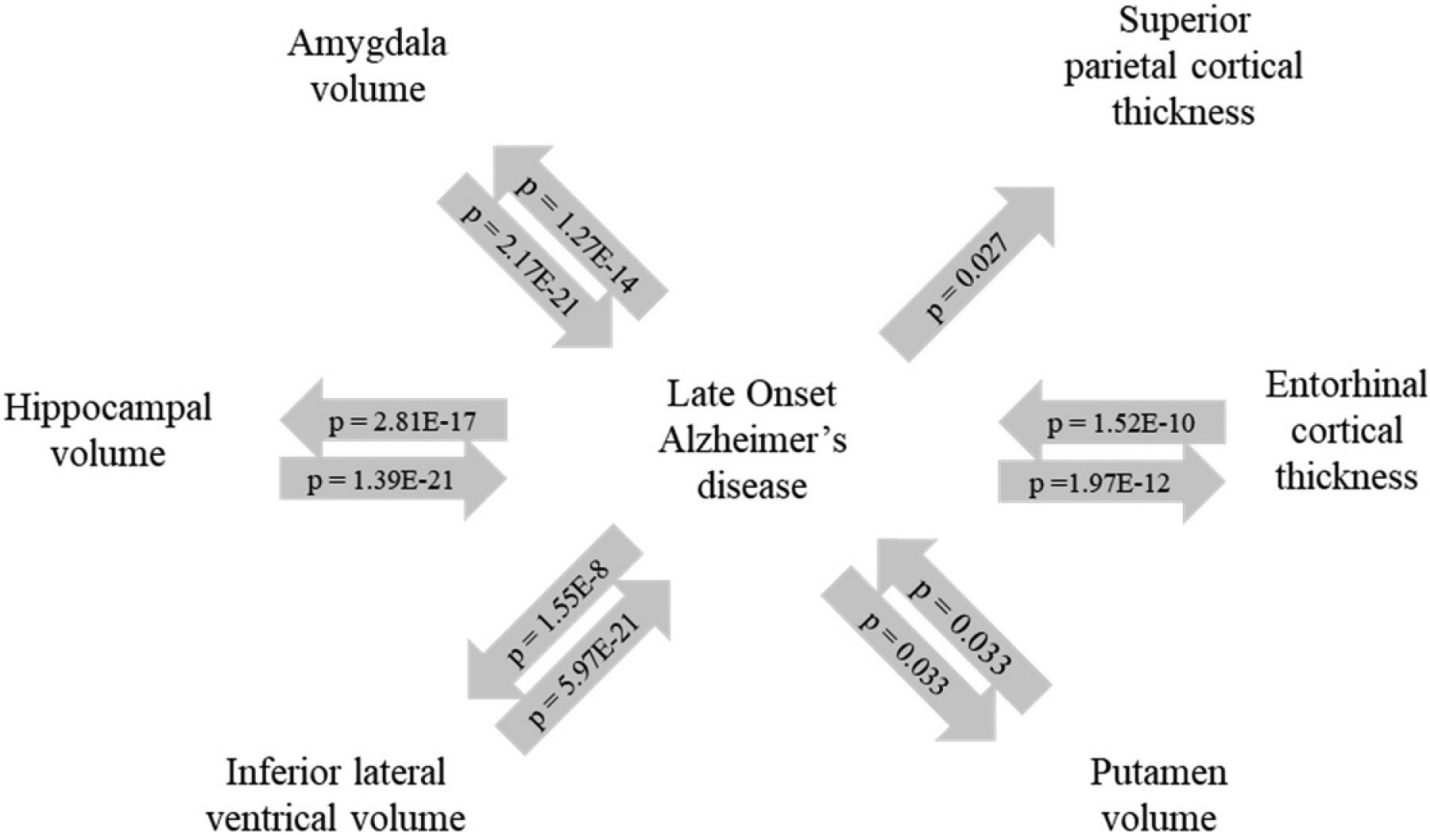
Results from the Mendelian randomization analysis between late-onset Alzheimer's disease and regional brain morphology. Arrows indicate the directionality of the causal relation, with the corresponding p-value corrected for multiple testing by FDR.

MR analysis was performed to assess the directionality of the associations between the mediating brain regions and LOAD. A significant bidirectional relationship was found between LOAD and the entorhinal cortex, amygdala, hippocampus and inferior lateral ventricle (Supplemental Table 5; Figure 3). All MR methods (IVW, weighted median and MR-Egger) resulted in a significant negative effect of the entorhinal cortex, amygdala and hippocampus on LOAD. The reverse MR also resulted in a significant negative effect of LOAD on these brain regions. The relationship between the inferior lateral ventricle with LOAD was significantly positive in both directions indicating that a larger volume of the inferior lateral ventricle increased the risk of LOAD and vice versa. Sensitivity analysis revealed violations of assumptions in some of the models. The MR-Egger estimate was unreliable for the effect of the entorhinal cortex and inferior lateral ventricle, hippocampus and amygdala (all I^2 ^< 80%) on LOAD. However, for each analysis, the results across methods were in agreement on the directionality of the effects, supporting the conclusions. For the relation between the putamen and LOAD, only the IVW method was significant in both directions. The model testing the effect of putamen volume on LOAD gave a significant Cochran's Q statistic (p < 0.001) indicating a potential violation of the instrumental variable assumption.

In addition, LOAD significantly increased the superior parietal cortex but not *vice versa*. A significant Cochran's Q statistic (p < 0.001) for this model indicates a possible violation of the instrumental variable assumption. Leave-one-out analysis showed that excluding either *APOE* ε4 or rs75627662 from the analysis resulted in a non-significant association. In addition, exclusion of rs7384878 lead to a reduction in the width of the confidence interval.

## Discussion

In this study, we aimed to determine whether effects of genetic variation on LOAD can be explained by regional brain morphology. To this aim, mediation analysis was performed using data collected by UKB and ADNI. We show a significant mediation effect of the entorhinal cortex, amygdala, hippocampus and inferior lateral ventricle on the relation between *APOE* ε4 and LOAD. In addition, the effects of rs75627662 and rs7384878 on LOAD were mediated by the hippocampus and superior parietal cortex, respectively. MR was subsequently used to determine the directionality of the relationship between LOAD and the mediating brain regions. These results indicated a bidirectional relationship between the entorhinal cortex, hippocampus, amygdala and lateral inferior ventricle, and LOAD.

We showed that *APOE* ε4 increased the risk of LOAD by altering the morphology of brain regions reported to be affected at an early stage in the pathological process of LOAD.^17,18,55,56^ Our results correspond with previous research on the association of *APOE* ε4 with the middle temporal lobe, more specifically the entorhinal cortex, amygdala and hippocampus.^27,57^ Mediation analysis has been applied previously to study the determinants of LOAD. These studies differed from the current study by the variables used. Clinical scores or measures for cognitive function and/or behavior were used as outcome measures and pathological hallmarks such as amyloid-β and tau were used as mediator and independent variables.^3,58–65^ Similar to our study, inclusion of brain measures as mediators identified the entorhinal cortex, hippocampus, amygdala and ventricles as mediators.^59,60,64,65^

MR pointed to a bidirectional effect between LOAD and the entorhinal cortex, hippocampus, amygdala, and the inferior lateral ventricle. From our results we can derive the following pathway regarding the effect of *APOE* ε4 on LOAD: The presence of *APOE* ε4 leads to a smaller entorhinal cortex, hippocampus and amygdala, and larger volume of the inferior lateral ventricles. These differences in brain morphology increase the risk of developing LOAD, indicating that the effect of *APOE* ε4 is at the start of the disease pathology. The bidirectionality we found indicates that LOAD may lead to aggravation of the degeneration of the entorhinal cortex, hippocampus and amygdala, and expansion of the inferior lateral ventricle. Multiple hypotheses have been raised regarding the pathological pathway by which *APOE* ε4 increases the risk of LOAD.^61^ APOE plays a fundamental role in brain functioning by facilitating neuronal cholesterol metabolism, which is essential for axonal growth and synapse formation.^34,66^ The APOE4 isoform was previously shown to lead to less efficient cholesterol transport and amyloid-β clearance, compared to the APOE2 and APOE3 isoforms.^67^ Accumulation of amyloid-β is one of the hallmarks of LOAD and plays a central role in the post-mortem diagnosis of LOAD and in many running theories regarding LOAD pathology.^4^

In addition to *APOE* ε4, rs75627662 and rs7384878 were significantly related to LOAD. The effect of rs75627662 was mediated by the entorhinal cortex, amygdala and hippocampus, and the effect of rs7384878 was mediated by the superior parietal cortex and putamen. Jansen et al. mapped rs75627662 to *APOE* and rs7384878 to *ZCWPW1.*^11^ The associations we found between rs75627662, the entorhinal cortex, amygdala and hippocampus, and LOAD might be a reflection of the relations we found for *APOE* ε4. *ZCWPW1* encodes for a histone modification reader, involved in epigenetic regulation.^68^ In addition, *ZCWPW1* is in high linkage disequilibrium (LD) with multiple other genes including *PILRB*, *ZNF3*, *RFX3* and *NYAP1*. The effect of *ZCWPW1* on LOAD is likely to be an indirect effect via epigenetic modification of target genes or a reflection of a gene in LD.^69^ *PILRB* was shown to be expressed in multiple brain regions and expression levels were lower in LOAD cases compared to controls.^70^ *PILRB* is involved in regulation of the immune response.^70^ *ZNF3* is involved in a pathway resulting in protein degradation, oxidative stress, cell cycle control and tau pathology.^71^ Another hypothesis is that *ZCWPW1* activates binding of RFX3, thereby suppressing insulin resistance and decreasing the risk of LOAD.^71^ Lastly, *NYAP1* was shown in mice to affect brain size, neurite elongation and neuronal morphology.^68^ MR showed that the relationship between LOAD and the superior parietal cortex was unidirectional, with a higher risk of LOAD leading to an increased thickness of the superior parietal cortex. However, the MR model of the superior parietal cortex might be violating the instrumental variable assumption, making the validity of these results questionable. Based on our results we hypothesize that *APOE* ε4 induces LOAD via lipid metabolism, amyloid-β accumulation and inflammation. Secondary processes including protein degradation, oxidative stress and tau pathology, mediated via *ZCWPW1,* could lead to the spreading of neurodegeneration to other brain regions.

Several study limitations must be acknowledged. We found multiple, biologically plausible pathways of mediation in ADNI that were not replicated in UKB. This discrepancy between the two cohorts has multiple possible explanations. Due to a lack of UKB participants with a diagnosis of LOAD and MRI brain measures, a LOAD-by-proxy score was used instead of the LOAD status based on clinical diagnosis that was used in ADNI. The validity of the LOAD-by-proxy score as a substitute for clinical LOAD might be questioned. The LOAD-by-proxy score was used previously in GWAS, which showed a high genetic correlation between standard case-control status and the UKB by-proxy phenotype.^11,12,28^ However, the current study only used a subset of UKB participants and the proxy score may not be an appropriate substitute for clinical LOAD status.^11,72^ Despite the high genetic correlation, true LOAD cases in the full UKB sample have higher polygenic risk scores compared to proxy cases^28^ and inconsistent results have been attributed to the use of the LOAD proxy score.^73^ The LOAD-by-proxy score is sensitive to recall bias, because the score is based on self-reported parental information by the participants. Additionally, parental LOAD status may neither strongly correspond to the clinical LOAD status of the participant^13,72,74,75^ nor indicate underlying pathophysiology. A second explanation for the differing results from ADNI and UKB is that these are different types of cohorts. ADNI is a case-control cohort in which LOAD cases were compared to controls that were generally healthy, predominantly of European White ancestry and overwhelmingly highly educated participants,^30,76^ with likely greater cognitive reserve (and thereby possibly delayed LOAD onset), thereby limiting the generalizability of the results. Substantial differences between ADNI and community based cohorts were reported previously.^76^ UKB participants have been shown to be overly healthy compared to the general population,^77^ raising the possibility of a higher resilience to the influence of genetic risk factors associated with LOAD. Additionally, UKB participants were on average slightly younger than the ADNI participants. Only participants that responded to the invitation for a second assessment underwent MRI, enhancing the possibility of healthy volunteer bias. Lastly, MRI measures from UKB were obtained using FreeSurfer version 5.3 whereas MRI scans from ADNI were processed by version 5.1. Different versions of FreeSurfer have been compared by others, who concluded that there were significant differences in the absolute values of the measures obtained.^78,79^ However, results from correlation studies as well as the ability to differentiate between cases and controls were comparable. It is therefore possible to compare the results obtained from UKB and ADNI.^78,79^ Another limitations of this study was the exclusion of participants with non-European white ancestry, limiting the generalizability of our results beyond this population. Lastly, we performed one sample MR for the entorhinal cortex, amygdala, hippocampus and inferior lateral ventricle using only on the ADNI sample. The sample size of this subgroup was relatively small and might have limited the statistical power of the analysis. Future studies should repeat the analysis, using a larger sample.

Our findings demonstrate that combining genetic and neuroimaging data can give insight into the causal neuropathological pathways of LOAD. Brain regions that were shown by MR to precede the diagnosis of LOAD (entorhinal cortex, amygdala, hippocampus and inferior lateral ventricle) could be potential targets for an early diagnosis and treatment or prevention of LOAD. Changes in brain regions occurring after the LOAD diagnosis (entorhinal cortex, amygdala, hippocampus, inferior lateral ventricle and superior parietal cortex) could be use to assess the progression of the disease as well as potential treatment targets. Differentiating between brain regions involved in the onset and progression of the disease can guide research into identifying the different pathological processes involved and multiple treatment targets for each stage of the disease. Future studies may expand the number of genetic risk variants under investigation or combine the effects of multiple SNPs into a polygenic risk score. Through this study we illustrated that mediation analysis combined with MR, enabling statements about causality, may increase knowledge on the (order of) pathological processes underlying LOAD. This can be valuable for developing tools for optimized diagnosis and treatment.

## Supplemental Material

sj-docx-2-alr-10.1177_25424823251328300 - Supplemental material for Brain morphology mediating the effects of common genetic risk variants on Alzheimer’s disease Supplemental material, sj-docx-2-alr-10.1177_25424823251328300 for Brain morphology mediating the effects of common genetic risk variants on Alzheimer’s disease by Esmee M Breddels, Yelyzaveta Snihirova, Ehsan Pishva, Sinan Gülöksüz, Gabriëlla AM Blokland, Jurjen Luykx, Ole A Andreassen, David EJ Linden, Dennis van der Meer and in Journal of Alzheimer's Disease Reports

sj-xlsx-3-alr-10.1177_25424823251328300 - Supplemental material for Brain morphology mediating the effects of common genetic risk variants on Alzheimer’s disease Supplemental material, sj-xlsx-3-alr-10.1177_25424823251328300 for Brain morphology mediating the effects of common genetic risk variants on Alzheimer’s disease by Esmee M Breddels, Yelyzaveta Snihirova, Ehsan Pishva, Sinan Gülöksüz, Gabriëlla AM Blokland, Jurjen Luykx, Ole A Andreassen, David EJ Linden, Dennis van der Meer and in Journal of Alzheimer's Disease Reports

sj-xlsx-4-alr-10.1177_25424823251328300 - Supplemental material for Brain morphology mediating the effects of common genetic risk variants on Alzheimer’s disease Supplemental material, sj-xlsx-4-alr-10.1177_25424823251328300 for Brain morphology mediating the effects of common genetic risk variants on Alzheimer’s disease by Esmee M Breddels, Yelyzaveta Snihirova, Ehsan Pishva, Sinan Gülöksüz, Gabriëlla AM Blokland, Jurjen Luykx, Ole A Andreassen, David EJ Linden, Dennis van der Meer and in Journal of Alzheimer's Disease Reports
