# Supplementary material for: Brain morphology mediating the effects of common genetic risk variants on Alzheimer’s disease
Source: J Alzheimers Dis Rep. 2025 Mar 24;9:25424823251328300. doi: 10.1177/25424823251328300 (PMC11938454; doi:10.1177/25424823251328300)
Supplement: sj-docx-2-alr-10.1177_25424823251328300 - Supplemental material for Brain morphology mediating the effects of common genetic risk variants on Alzheimer’s disease [file sj-docx-2-alr-10.1177_25424823251328300.docx]

Supplemental Material

Brain morphology mediating the effects of common genetic risk variants on Alzheimer’s disease

Supplement A: LOAD proxy score.

A LOAD proxy score was used to assess the risk of LOAD in the UK Biobank sample. The LOAD proxy score was based on the number of parents diagnosed with LOAD and the age (at death) of the parent in the absence of a LOAD diagnosis. The R code used to create this score is shown below.

If a parent had a diagnosis of LOAD, they would contribute 1 to the LOAD proxy score. Parents without a diagnosis of LOAD would contribute between 0-0.32 to the LOAD proxy score, depending on their age (at death). This factor was created to account for the probability that the parent might still develop LOAD. If neither parent had a diagnosis, the participant would receive a LOAD proxy score between 0-0.64. One affected parent and one unaffected parent leads to a LOAD proxy score between 1-1.32 and two affected parents would therefore lead to a LOAD proxy score of 2.

# Calculate maternal and paternal risk scores

for(i in 1:dim(adps)[1]){

if (adps$ad.fat[i]==TRUE){adps$rsf[i] <- 1

} else if (is.na(adps$age.fat[i])|adps$age.fat[i] <= 0){adps$rsf[i] <- NA

} else {adps$rsf[i] <- max(min(((100-adps$age.fat[i])/100),0.32),0)}

if (adps$ad.mot[i]==TRUE){adps$rsm[i] <- 1

} else if (is.na(adps$age.mot[i])|adps$age.mot[i] <=0){adps$rsm[i] <- NA

} else {adps$rsm[i] <- max(min(((100-adps$age.mot[i])/100),0.32),0)}

}

## Calculate AD proxy score

adps$ADps <- NULL

for(i in 1:dim(adps)[1]){

if (adps$ad[i]==TRUE){adps$ADps[i] <- 2

} else {adps$ADps[i] <- adps$rsf[i] + adps$rsm[i]}

}

The LOAD proxy score can get any value from 0-0.64, 1-1.32 or 2. The variable is therefore not normally distributed (Figure A1). We performed a simulation with 100,000 replications to determine if the alpha of 0.05 was preserved. The R code for this simulation is shown below. The simulation resulted in 5% of the simulations resulting in a p-value smaller or equal to 0.05, therefore we concluded that the alpha was preserved and linear regression analysis was appropriate to use with the LOAD proxy score as outcome.


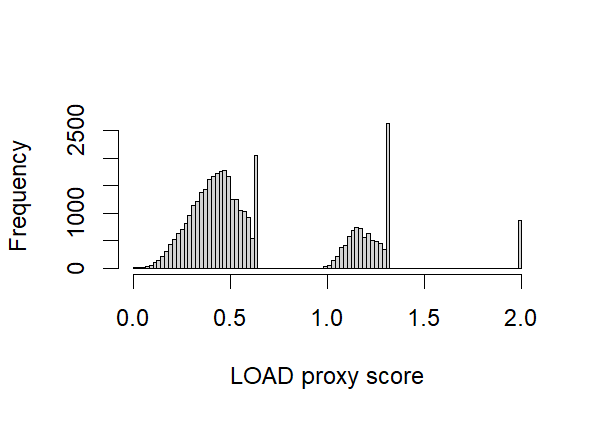


**Figure A1.** Histogram of the distribution of the LOAD proxy score in the UKB sample.

y <- data$ADps

n <- length(y)

iters <- 100000

pval <- rep(NA,iters)

for(i in 1:iters){

x <- rnorm(n)

res <- lm(y~x)

pval[i] <- coef(summary(res))[2,4]

}

hist(pval)

mean(pval <= 0.05)

Supplemental Table 1a. Description of the dependent and independent variables selected from UK Biobank, including their corresponding field codes.

| **Variable** | **Description** | **Field code UK Biobank** |
| --- | --- | --- |
| **AD proxy score** |  |  |
| Participant AD diagnosis | Based on presence of ICD-10 codes G30 or F00, or report of AD. | 41270, 42020 |
| Paternal AD diagnosis | Self-reported by participants. | 20107 |
| Paternal age (at death) | Reported by participant. | 29,461,807 |
| Maternal AD diagnosis | Self-reported by participants. | 20110 |
| Maternal age (at death) | Reported by participant. | 18,453,526 |
| **Demographics** |  |  |
| Age | Age filled in at the time of assessment 2 (visit when imaging data was collected) | 21003 |
| Sex | Based on the sex based on genotyping. |  |
| Ancestry | Self-identified as 'White British' and very similar genetic ancestry based on a principal components analysis of the genotypes. | 22006 |
| Site | UK Biobank assessment centre | 54 |
| **Brain measures** |  |  |
| Cortical area | Freesurfer DKT | 27143-27173 |
|  |  | 27236-27266 |
|  | Freesurfer desikan white | 26721, 26822, |
|  |  | 26752, 26853, |
|  |  | 26722, 26823 |
|  | Freesurfer a2009s | 27371, 27593 |
| Cortical thickness | Freesurfer DKT | 27174-27204 |
|  |  | 27267-27297 |
|  | Freesurfer desikan white | 26755, 26856, |
|  |  | 26786, 26887, |
|  |  | 26756, 26857 |
|  | Freesurfer a2009s | 27445, 27667 |
| Subcortical volume | Freesurfer ASEG | 26514-26537 |
|  |  | 26552-26567 |
|  |  | 26583-26598 |

Supplemental Table 1b. Description of the dependent and independent variables selected from ADNI. ADNI data was collected in multiple waves, for each wave, the table containing the needed variables and their corresponding field codes are presented.

| **Variable** | **Description** | **Table ADNI** | **Field code ADNI** |
| --- | --- | --- | --- |
| **AD** |  |  |  |
| Diagnosis ADNI1 | 1=NL; 2=MCI; 3=AD | DXSUM | DXCURREN |
| Diagnosis ADNIGO/2 | 1=Stable: NL to NL; 2=Stable: MCI to MCI; 3=Stable: Dementia to Dementia; 4=Conversion: NL to MCI; 5=Conversion: MCI to Dementia; 6=Conversion: NL to Dementia; 7=Reversion: MCI to NL; 8=Reversion: Dementia to MCI; 9=Reversion: Dementia to NL |  | DXCHANGE |
|  |  |  |  |
|  |  |  |  |
|  |  |  |  |
|  |  |  |  |
| **Demographics** |  |  |  |
| Year of birth | Participant year of birth | PTDEMOG | PTDOBYY |
| Date of imaging | Examination Date | UCSFFSX51 | EXAMDATE |
| Sex/gender | 1=Male; 2=Female | PTDEMOG | PTGENDER |
| Ancestry | 1=American Indian or Alaskan Native; 2=Asian; 3=Native Hawaiian or Other Pacific Islander; 4=Black or African American; **5=White**; 6=More than one race; 7=Unknown | PTDEMOG | PTRACCAT |
| Site |  |  | SITEID |
| **Brain measures** |  |  |  |
| Cortical area | ADNI1/GO/2: 3T  (Freesurfer 5.1) | UCSFFSX51 | ST13SA-ST15SA, ST23SA-ST26SA, ST31SA, ST32SA, ST34SA-ST36SA, ST38SA-ST40SA, ST43SA-ST52SA, ST54SA-ST60SA, ST62SA, ST72SA-ST74SA, ST82SA-ST85SA, ST90SA, ST91SA, ST93SA-ST95SA, ST97SA-ST99SA, ST102SA-ST111SA, ST113SA-ST119SA, ST121SA, ST129SA, ST130SA |
| Cortical thickness | ADNI1/GO/2: 3T  (Freesurfer 5.1) | UCSFFSX51 | ST13TA-ST15TA, ST23TA-ST26TA, ST31TA, ST32TA, ST34TA-ST36TA, ST38TA-ST40TA, ST43TA-ST52TA, ST54TA-ST60TA, ST62TA, ST72TA-ST74TA, ST82TA-ST85TA, ST90TA, ST91TA, ST93TA-ST95TA, ST97TA-ST99TA, ST102TA-ST111TA, ST113TA-ST119TA, ST121TA, ST129TA, ST130TA |
| Subcortical volume | ADNI1/GO/2: 3T,  (Freesurfer 5.1) | UCSFFSX51 | ST1SV-ST9SV, ST11SV, ST12SV, ST16SV-ST18SV, ST21SV, ST29SV, ST30SV, ST37SV, ST42SV, ST53SV, ST61SV, ST65SV, ST68SV-ST71SV, ST75SV-ST77SV, ST80SV, ST88SV, ST89SV, ST96SV, ST101SV, ST112SV, ST120SV, ST124SV, ST125SV, ST127SV, ST128SV, ST147SV, ST148SV, ST150SV, ST151SV, ST153SV-ST155SV |

Supplemental Table 2. Lead SNPs for each genomic risk loci obtained by FUMA. The uniqID is the unique ID of the SNPs consisting of chromosome:position:allele1:allele2 where alleles are alphabetically ordered. The rsID of the top lead SNP is based on dbSNP build 146. The SNP is located on the chromosome in the column “chr” at position “pos” on hg19 ranging from “start” to “end”. Not all participants had information on all genetic variants, participants were excluded in a model-based manner based on the variant included, resulting in the samples sizes below.

|  | **uniqID** | **rsID** | **chr** | **pos** | **start** | **end** | **UKB sample size** |
| --- | --- | --- | --- | --- | --- | --- | --- |
| 1 | 1:207750568:C:T | rs679515 | 1 | 207750568 | 207679307 | 207806730 | 38,845 |
| 2 | 2:127891427:A:C | rs4663105 | 2 | 127891427 | 127826533 | 127894851 | 37,728 |
| 3 | 3:57226150:C:T | rs184384746 | 3 | 57226150 | 56252241 | 57879720 | N/A |
| 4 | 4:11024682:C:G | rs6448451 | 4 | 11024682 | 11014822 | 11041549 | 38,897 |
| 5 | 6:40942196:A:G | rs187370608 | 6 | 40942196 | 40706366 | 41129252 | N/A |
| 6 | 7:99932049:C:T | rs7384878 | 7 | 99932049 | 99777422 | 100091795 | 38,325 |
| 7 | 7:145950029:C:T | rs114360492 | 7 | 145950029 | 145181183 | 146573693 | N/A |
| 8 | 8:27466315:C:T | rs1532278 | 8 | 27466315 | 27456253 | 27468503 | 38,922 |
| 9 | 11:60021948:A:G | rs1582763 | 11 | 60021948 | 59826677 | 60099912 | 38,853 |
| 10 | 11:85850243:C:T | rs3844143 | 11 | 85850243 | 85652251 | 85869737 | 38,922 |
| 11 | 17:4984447:A:G | rs9916042 | 17 | 4984447 | 4958842 | 5013491 | 38,508 |
| 12 | 19:1053524:C:G | rs3752241 | 19 | 1053524 | 1053524 | 1053524 | 38,176 |
| 13 | 19:45413576:C:T | rs75627662 | 19 | 45413576 | 44724661 | 46491516 | 38,771 |

N/A: no participant had information available for this snp

Supplemental Table 3. Total effect of the genetic variants on the LOAD proxy score (UK Biobank) and the odds of LOAD (ADNI), corrected for age and sex. For the ADNI cohort, the estimates were additionally transformed into the odds ratio (OR) and presented in the table below.

|  | **UK Biobank** | | **ADNI** | | | |
| --- | --- | --- | --- | --- | --- | --- |
| **Genetic variant** | **Estimate** | **p** | **Estimate** | **OR** | **p** |  |
| APOE4 | 0.105 | 8.97E-146 | 1.339 | 3.817 | 9.85E-15 |  |
| rs75627662 | 0.050 | 4.96E-43 | 0.736 | 2.088 | 2.48E-06 |  |
| rs4663105 | 0.011 | 2.36E-04 | 0.159 | 1.172 | 0.221 |  |
| rs7384878 | -0.011 | 0.001 | -0.392 | 0.676 | 0.009 |  |
| rs679515 | 0.012 | 0.002 | 0.087 | 1.091 | 0.605 |  |
| rs1582763 | -0.009 | 0.002 | -0.144 | 0.866 | 0.275 |  |
| rs1532278 | -0.009 | 0.003 | -0.246 | 0.782 | 0.083 |  |
| rs3844143 | -0.009 | 0.003 | -0.057 | 0.944 | 0.671 |  |
| rs6448451 | 0.006 | 0.082 | 0.139 | 1.149 | 0.341 |  |
| rs9916042 | 0.003 | 0.317 | 0.291 | 1.337 | 0.040 |  |
| rs3752241 | -0.002 | 0.626 | -0.110 | 0.896 | 0.532 |  |

**Supplemental Table 4.** Results from all mediation analysis performed. Analyses were performed separately for UK Biobank and ADNI. The mediator model (path a) represents the effect of the genetic variant on the brain measure. The mediation effect (a*b) is the indirect of mediation effect of the brain measure on the association between the variant and LOAD. *indicates the p-value is smaller than the FDR threshold, thus significant. **See Excel file.**

Supplemental Table 5a. Results of the Mendelian randomization analysis. The direction indicates whether the mendelian randomization was performed from the brain measure to LOAD (→) or reverse (←). Multiple methods were used and presented: IVW (Inverse-variance weighted), MR-Egger and weighted median. SE = standard error; UKB = UK Biobank.

| **Brain measure** | **Direction** | **IVW** | | | | **MR-Egger** | | | | **Weighted median** | | | |
| --- | --- | --- | --- | --- | --- | --- | --- | --- | --- | --- | --- | --- | --- |
|  |  | **Beta** | **SE** | **p** | **Beta** | | **SE** | **p** | **Beta** | | **SE** | **p** |  |
| Entorhinal thickness | → | -3.456 | 0.533 | **8.89E-11** | -3.994 | | 0.598 | **2.32E-11** | -3.542 | | 0.764 | **3.51E-06** |  |
|  | ← | -0.239 | 0.033 | **9.85E-13** | -0.267 | | 0.046 | **4.65E-09** | -0.256 | | 0.050 | **3.04E-07** |  |
| Amygdala | → | -3.088 | 0.319 | **3.62E-22** | -3.369 | | 0.439 | **1.69E-14** | -3.219 | | 0.614 | **1.59E-07** |  |
|  | ← | -0.303 | 0.039 | **5.28E-15** | -0.303 | | 0.053 | **8.60E-09** | -0.307 | | 0.058 | **1.12E-07** |  |
| Hippocampus | → | -2.870 | 0.293 | **1.16E-22** | -2.774 | | 0.394 | **1.82E-12** | -3.001 | | 0.547 | **4.02E-08** |  |
|  | ← | -0.330 | 0.038 | **9.38E-18** | -0.327 | | 0.052 | **4.33E-10** | -0.323 | | 0.060 | **5.95E-08** |  |
| Inferior Lateral Ventricle | → | 3.993 | 0.419 | **1.49E-21** | 3.942 | | 0.590 | **2.40E-11** | 4.219 | | 1.051 | **0.0001** |  |
|  | ← | 0.228 | 0.040 | **1.04E-08** | 0.224 | | 0.054 | **3.51E-05** | 0.232 | | 0.054 | **1.71E-05** |  |
| Putamen | → | -2.999 | 1.402 | **0.032** | -0.242 | | 0.137 | 0.078 | -1.706 | | 3.747 | 0.649 |  |
|  | ← | -0.096 | 0.045 | **0.033** | -0.041 | | 0.029 | 0.156 | -0.058 | | 0.046 | 0.213 |  |
| Superior parietal (UKB) | → | 1.746 | 0.759 | **0.021** | 2.184 | | 1.298 | 0.092 | 0.852 | | 0.556 | 0.126 |  |
|  | ← | 0.198 | 0.087 | **0.022** | 0.240 | | 0.108 | **0.027** | 0.221 | | 0.046 | **1.28E-06** |  |

Supplemental Table 5b. Results of the diagnostics tests of the Mendelian randomization analysis. The direction indicates whether the mendelian randomization was performed from the brain measure to LOAD (→) or reverse (←). Multiple diagnostics were performed and presented.

| **Brain measure** | **Direction** | **MR-Egger (intercept)** | | | | **I²-statistic** | | **Cochran's Q test** | |
| --- | --- | --- | --- | --- | --- | --- | --- | --- | --- |
|  |  | Beta | SE | p |  | | Estimate | | p |
| Entorhinal thickness | → | 0.112 | 0.069 | 0.108 | 0.728 | | 15.021 | | 0.090 |
|  | ← | 0.021 | 0.023 | 0.361 | 0.818 | | 8.311 | | 0.503 |
| Amygdala | → | 0.057 | 0.061 | 0.352 | 0.667 | | 6.060 | | 0.734 |
|  | ← | 0.000 | 0.027 | 0.987 | 0.819 | | 3.328 | | 0.950 |
| Hippocampus | → | -0.022 | 0.060 | 0.717 | 0.729 | | 4.538 | | 0.873 |
|  | ← | -0.002 | 0.027 | 0.931 | 0.819 | | 2.771 | | 0.973 |
| Inferior Lateral Ventricle | → | 0.008 | 0.063 | 0.898 | 0.438 | | 9.714 | | 0.374 |
|  | ← | 0.003 | 0.027 | 0.918 | 0.817 | | 2.707 | | 0.975 |
| Putamen | → | -0.242 | 0.137 | 0.078 | 0.080 | | 51.321 | | **6.07E-08** |
|  | ← | -0.041 | 0.029 | 0.156 | 0.815 | | 10.650 | | 0.300 |
| Superior parietal (UKB) | → | -0.006 | 0.013 | 0.670 | 0.562 | | 591.96 | | **1.11E-121** |
|  | ← | -0.002 | 0.003 | 0.497 | 0.983 | | 39.381 | | **9.83E-06** |

**Supplemental Table 6.** Results of the meta-analysis combining the mediation results from UK Biobank and ADNI. The estimate, SE (standard error) and p-value of the meta-analysis per genetic variant and brain measure. p-values were corrected for multiple testing by FDR. Cochran's Q-statistic and I2 were used to determine the heterogeneity. **See Excel file.**

N = 505,502 UKB participants

N = 1740 ADNI 1/GO/2 participants

n = 39,565 White British ancestry

5,984 excluded

n = 780 White ancestry

9 excluded

n = 38,922 with parental LOAD status and age (at death)

643 excluded

n = 476 LOAD or cognitively normal

304 excluded

n = 46,852 with MRI data

465,447 excluded

n = 1068 with MRI data

672 excluded

n = 45,549 were genotyped and had information on at least one SNPs of interest

1,303 excluded

n = 789 were genotyped and had information on the SNPs of interest

279 excluded

Supplemental Figure 1. Overview of the inclusion and exclusion of UK Biobank and ADNI participants.

SNP

Brain morphology

Global measure

Sex

Age

LOAD proxy score

Supplemental Figure 2. Directed acyclic graph (DAG) for the selection of covariates. Age has an effect on both brain morphology and the LOAD proxy score and is considered a confounder and should be included as a covariate in the models. Sex and the different global measures (total surface area, global mean thickness or total intracranial volume) are related to the measure of brain morphology, inclusion as a covariate will reduce overall variation and improve precision.


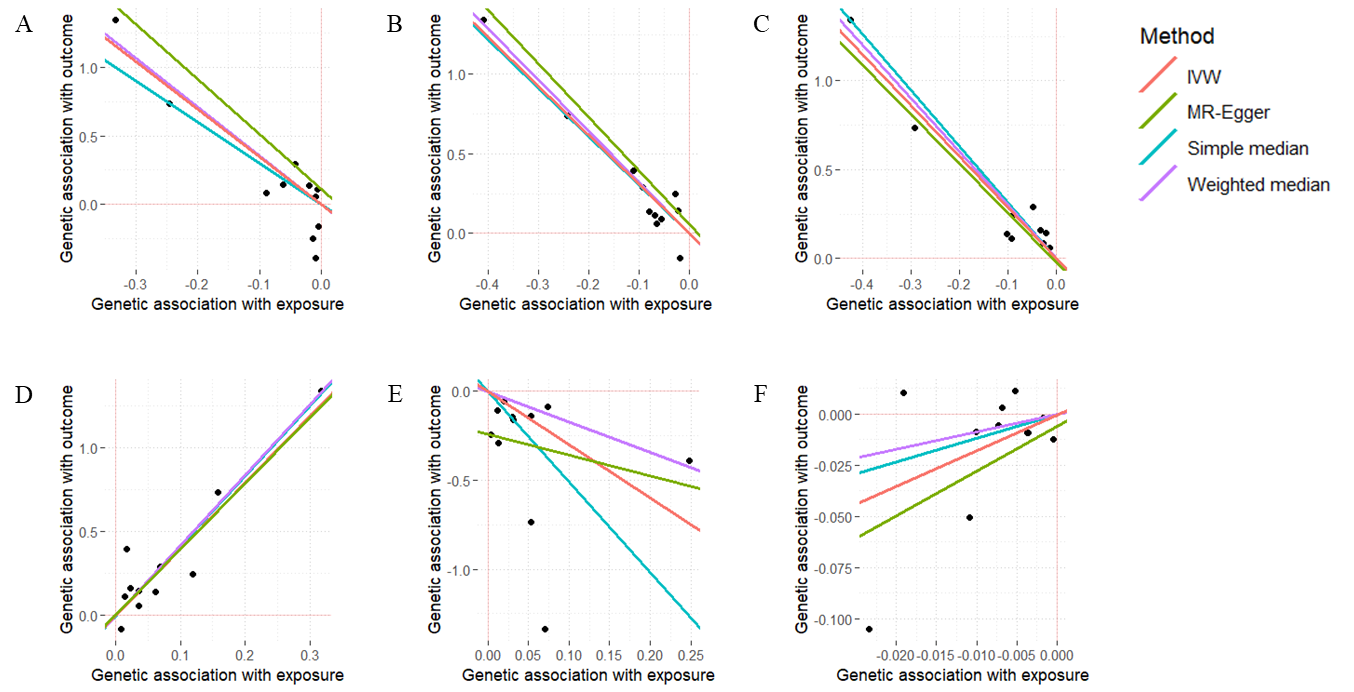


Supplemental Figure 3a. Diagnostic plots of the Mendelian randomization (MR). Plots show the genetic association with the outcome and the exposure calculated using the different methods. (A) Entorhinal cortex, (B) Amygdala, (C) Hippocampus, (D) Inferior lateral ventricle, (E) Putamen, (F) Superior parietal cortex.


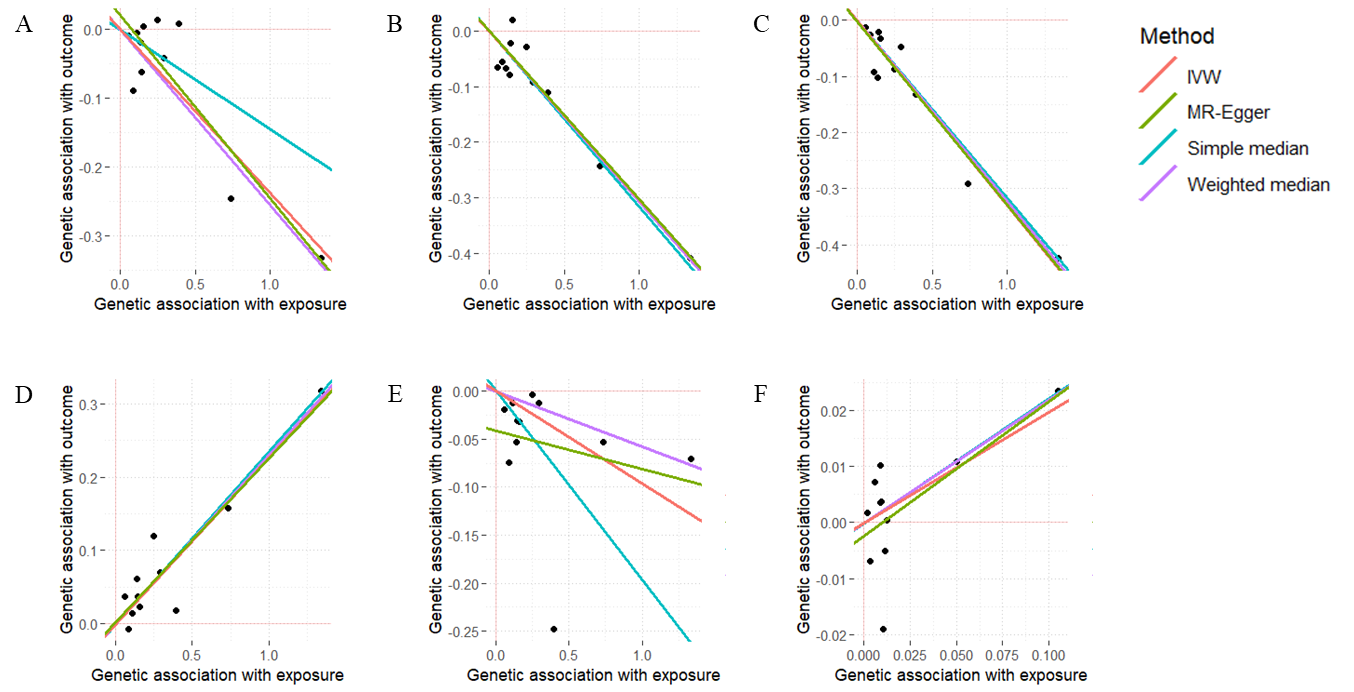


Supplemental Figure 3b. Diagnostic plots of the reverse Mendelian randomization (MR). Plots show the genetic association with the outcome and the exposure calculated using the different methods. (A) Entorhinal cortex, (B) Amygdala, (C) Hippocampus, (D) Inferior lateral ventricle, (E) Putamen, (F) Superior parietal cortex.


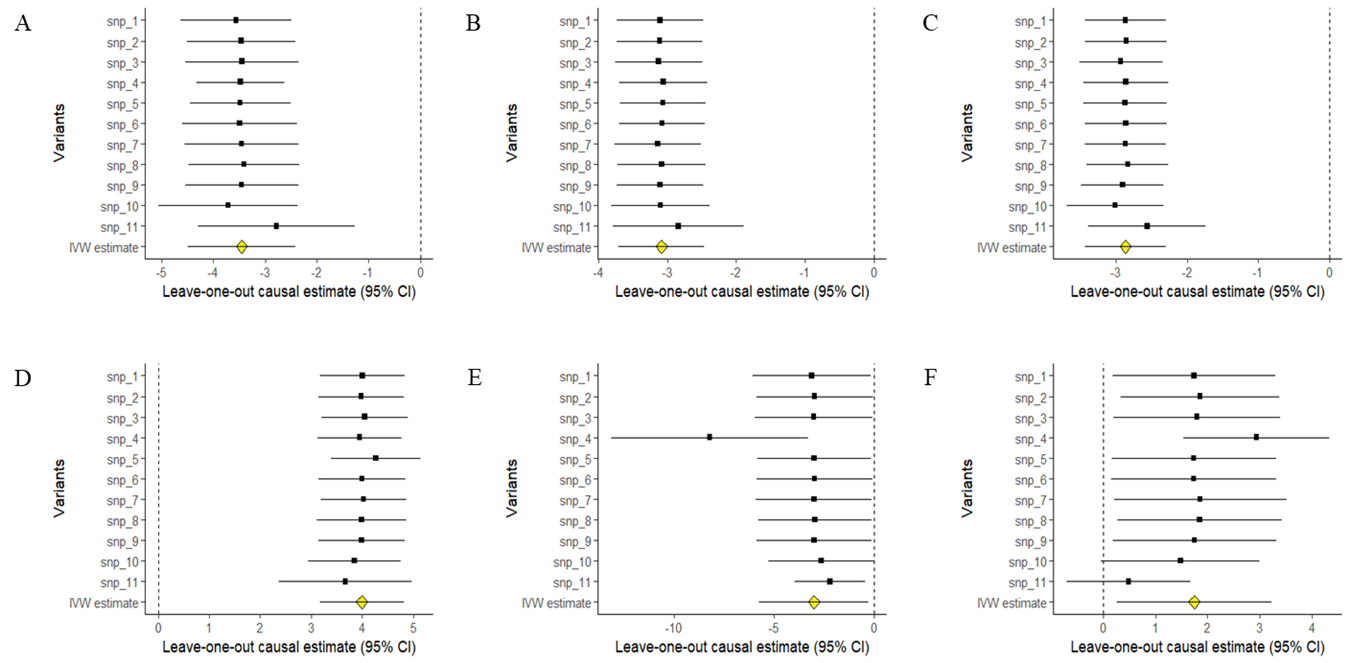


Supplemental Figure 3c. Diagnostic plots of the Mendelian randomization (MR) analysis using leave-one-out analysis. Plots show the estimates and 95% CI from the leave-one-out analysis compared to the overall IVW estimate. (A) Entorhinal cortex, (B) Amygdala, (C) Hippocampus, (D) Inferior lateral ventricle, (E) Putamen, (F) Superior parietal cortex.


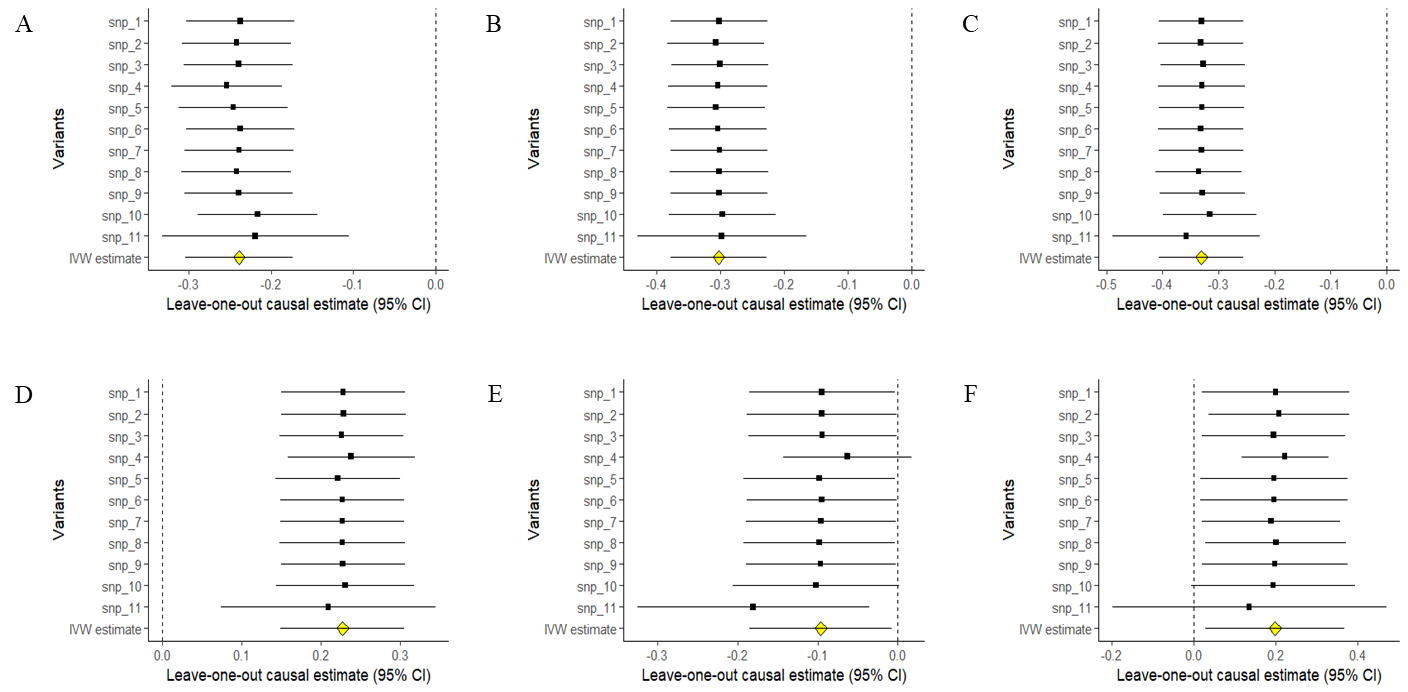


Supplemental Figure 3d. Diagnostic plots of the reverse Mendelian randomization (MR) analysis using leave-one-out analysis. Plots show the estimates and 95% CI from the leave-one-out analysis compared to the overall IVW estimate. (A) Entorhinal cortex, (B) Amygdala, (C) Hippocampus, (D) Inferior lateral ventricle, (E) Putamen, (F) Superior parietal cortex.
